# An Improved Ras Sensor for Highly Sensitive and Quantitative FRET-FLIM Imaging

**DOI:** 10.1371/journal.pone.0052874

**Published:** 2013-01-14

**Authors:** Ana F. Oliveira, Ryohei Yasuda

**Affiliations:** 1 Department of Neurobiology, Duke University Medical Center, Durham, North Carolina, United States of America; 2 Doctoral Program in Biomedicine and Experimental Biology, Center for Neuroscience and Cell Biology, University of Coimbra, Coimbra, Portugal; 3 Howard Hughes Medical Institute, Duke University Medical Center, Durham, North Carolina, United States of America; 4 Max-Planck Florida Institute, Jupiter, Florida, United States of America; Cornell University, United States of America

## Abstract

Ras is a signaling protein involved in a variety of cellular processes. Hence, studying Ras signaling with high spatiotemporal resolution is crucial to understanding the roles of Ras in many important cellular functions. Previously, fluorescence lifetime imaging (FLIM) of fluorescent resonance energy transfer (FRET)-based Ras activity sensors, FRas and FRas-F, have been demonstrated to be useful for measuring the spatiotemporal dynamics of Ras signaling in subcellular micro-compartments. However the predominantly nuclear localization of the sensors' acceptor has limited its sensitivity. Here, we have overcome this limitation and developed two variants of the existing FRas sensor with different affinities: FRas2-F (K_d_∼1.7 µM) and FRas2-M (K_d_∼0.5 µM). We demonstrate that, under 2-photon fluorescence lifetime imaging microscopy, FRas2 sensors provide higher sensitivity compared to previous sensors in 293T cells and neurons.

## Introduction

Ras is a member of a large family of small GTPase proteins that bind to and hydrolyze guanosine triphosphate (GTP) into guanosine diphosphate (GDP) [1]. Major subtypes include H-, N- and K-Ras, and all of these subtypes express ubiquitously [1]. Ras is important in transducing a wide range of extracellular signals from membrane receptors to intracellular signaling cascades [2], [3] that regulate many cellular processes, including cell cycle progression, differentiation and survival [3], [4]. When bound to GTP, Ras is active and able to bind and activate downstream effectors; whereas when bound to GDP, it is inactive [5]. In neurons, Ras plays critical roles in synaptic plasticity, neuronal morphogenesis, and learning and memory [1], [6]–[8]. Tight spatiotemporal regulation of Ras activity is central to the activation of specific signaling pathways in order to achieve appropriate biological outcomes [2], [9]. Therefore, it is crucial to measure the spatiotemporal dynamics of Ras signaling to understand how it regulates its diverse downstream targets.

In order to image intracellular signaling activity, many sensors based on FRET have been developed [10]. Because FRET strongly depends on the distance between the donor and acceptor, FRET can be used as a readout of protein-protein interactions for proteins fused to fluorophores [11]. A FRET based sensor called “Ras and interacting protein chimeric unit” (Raichu) enabled the measurement of the spatiotemporal dynamics of Ras activity in live cells [12]. Raichu consists of a fusion of enhanced yellow fluorescent protein (EYFP), HRas without the CAAX membrane targeting sequence, Ras binding domain of Raf1 (RBD), enhanced cyan fluorescent protein (ECFP), and KRas CAAX in a single polypeptide [12]. In the inactive GDP-bound form, ECFP and EYFP are located away from each other, thereby resulting in low FRET. Following Ras activation, RBD associates with active Ras. This brings ECFP and EYFP into close proximity, thereby increasing FRET [12].

**Figure 1:**
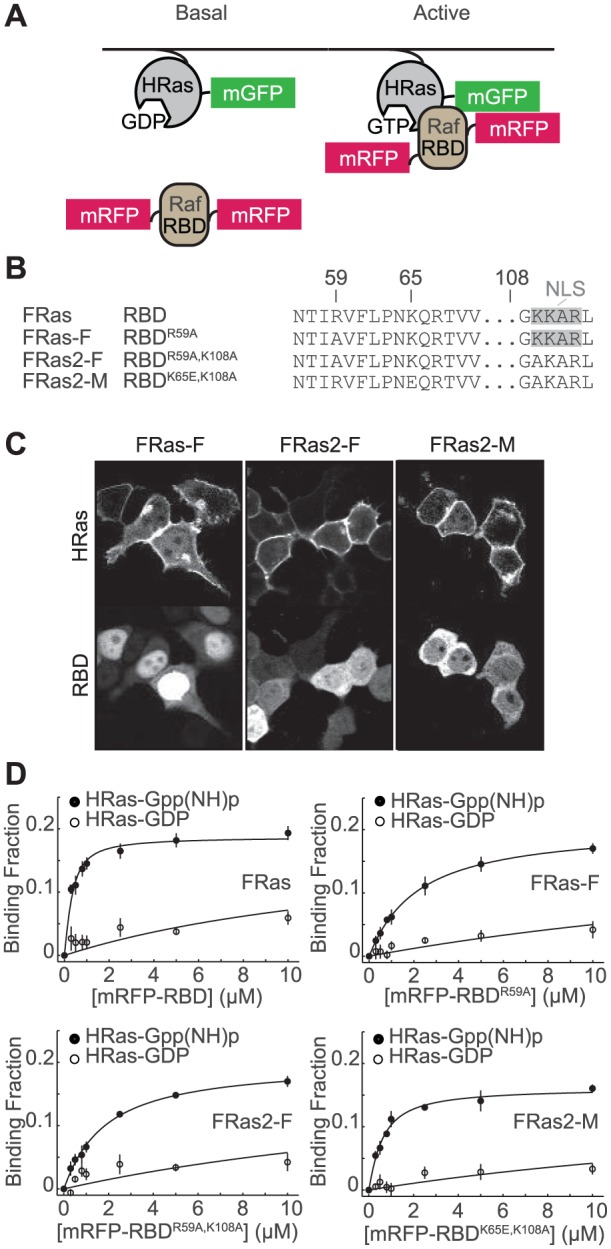
Design of Ras FRET sensors. (A) Schematics of Ras FRET sensors. mEGFP was tagged to the N terminus of HRas, and mRFP was attached to both termini of Ras binding domain of Raf1 (RBD), which binds selectively to active Ras. When HRas is activated, it binds to RBD, increasing FRET between mEGFP and mRFP. (B) Abbreviated amino acid sequence of RBD (WT), RBD^R59A^, RBD^R59A,K108A^ and RBD^K65E,K108A^, highlighting the mutations introduced in the RBD sequence to produce different Ras sensor acceptors. (C) Distribution of donor and acceptor for FRas-F, FRas2-F, and FRas2-M expressed in 293T cells. (D) Measurements of the affinity between sfGFP-tagged HRas and mRFP-tagged RBD. The binding fraction was measured with 2pFLIM as a function of the RBD concentration (average of 3–5 independent experiments). The dissociation constants between HRas and RBD mutations are summarized in Table 1.

Recently, a more sensitive imaging method based on FLIM in combination with a FRET-based Ras sensor optimized for FLIM, FRas, has been developed [13]. With this sensor, signaling activity in small subcellular compartments was quantified [13]. FRas is made of two components: HRas tagged with monomeric enhanced green fluorescent protein (mEGFP-HRas) and RBD tagged on each end with monomeric red fluorescent proteins (mRFP-RBD-mRFP) (Figure 1A). When mEGFP-Ras is activated, mRFP-RBD-mRFP binds to mEGFP-HRas, increasing FRET [13]. While FRas provided high sensitivity, the high affinity between HRas and RBD slowed Ras inactivation by inhibiting its interaction with GTPase-activating proteins (GAPs) [13]. To address this problem, a mutation (R59A) was introduced in RBD to decrease the affinity between Ras and RBD to create FRas-F, the Ras sensor with fast inactivation kinetics [13], [14].

While it has been demonstrated that the sensitivity of FRas-F is sufficiently high for imaging Ras signaling in small subcellular compartments such as dendritic spines of pyramidal neurons [13], we found that the acceptor of the sensor (mRFP-RBD^R59A^-mRFP) accumulates in the nucleus, limiting the sensitivity of the Ras sensor. In this paper, we identify the sequence in RBD that causes nuclear localization and introduce a point mutation to generate a new sensor, FRas2-F, which overcomes this problem. In addition, we develop a FRas2 variant, FRas2-M, with slightly increased RBD affinity. The new sensors show significantly improved sensitivity over previous sensors in both 293T cells and neurons.

## Materials and Methods

### DNA Constructs

The constructs pCI-mEGFP-HRas, pCI-mRFP-RBD-mRFP and pCI-mRFP-RBD^R59A^-mRFP were previously described [13]. Point mutations were introduced in the original pCI-mRFP-RBD^R59A^-mRFP or pCI-mRFP-RBD-mRFP to produce pCI-mRFP-RBD^R59A,K108A^-mRFP and pCI-mRFP-RBD^K65E,K108A^-mRFP, respectively.

### Protein Purification

Polyhistidine (His_6_)-superfolder GFP (sfGFP)-HRas without the C-terminal CAAX membrane targeting sequence [15] and His_6_-mRFP–RBD mutants were cloned into the pET bacterial expression vector. Proteins were overexpressed in *Escherichia coli* (BL21(DE3)pLysS), purified with Ni^+^-nitrilotriacetate (NTA) column (HisTrap, GE Healthcare) and desalted on a desalting column (PD10, GE Healthcare) equilibrated with 50 mM Tris-HCl (pH 8), 100 mM NaCl, 3 mM MgCl_2_ and 1 mM dithiothreitol (DTT). The concentration of the purified protein was measured by the absorbance of the fluorophore (sfGFP, *A*_489 nm_ = 83,000 cm^−1^ M^−1^ [16]; mRFP, *A*_584 nm_ = 50,000 cm^−1^ M^−1^ [17]).

### Measurements of the Affinity Between HRas and RBD

Purified sfGFP-HRas (without the CAAX membrane targeting sequence) was loaded with 25-fold molar excess of 2′,3′-O-*N*-methyl anthraniloyl–GppNHp (Gpp(NH)p) and GDP by incubating in the presence of 15 mM ethylenediaminetetraacetic acid (EDTA) for 30 min at 37°C. Then, 10 mM MgCl_2_ was added to the reaction [18]. The excess amount of Gpp(NH)p and GDP was removed with a desalting column (GE Healthcare). SfGFP-HRas and mRFP-RBD (or its mutants) were mixed and incubated at room temperature for 30 min. FRET between sfGFP and mRFP was quantified with fluorescence lifetime measurement. To obtain the fluorescence lifetime of free sfGFP-HRas, its fluorescence lifetime decay was fit with a single exponential function convolved with the Gaussian pulse response function:
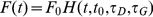
(1)where 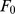 is the constant, and
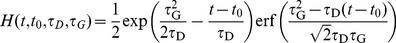
(2)in which 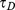 is the fluorescence lifetime of the free donor, 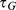 is the width of the Guassian pulse response function, 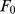 is the peak fluorescence before convolution, 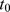 is the time offset, and 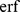 is the error function. To measure the fraction of donor bound to acceptor, the fluorescence lifetime decay was fit with a double exponential function convolved with the Gaussian pulse response function:
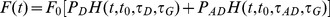
(3)where 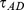 is the fluorescence lifetime of donor bound with acceptor and 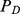 and 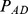 are the fraction of free donor and donor bound with acceptor, respectively. We fixed 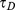 to the fluorescence lifetime obtained from free sfGFP-HRas.

The dissociation constant (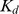) was obtained by fitting the relationship between the binding fraction (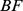) and the concentration of mRFP–RBD with the following equation:
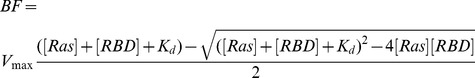
(4)


### Cell Culture and Transfection

293T cells (ATCC #: CRL-11268) were cultured in Dubelco's modified eagle medium (DMEM) supplemented with 10% fetal bovine serum (FBS) at 37°C in 5% CO_2_, and transfected with plasmids using Lipofectamine 2000 (Invitrogen). Approximately 16–18 hours after transfection, the medium was replaced with DMEM with low FBS (0.5%) for 8 hours, and subjected to imaging in a solution containing 30 mM HEPES (pH 7.3), 130 mM NaCl, 2.5 mM KCl, 1 mM CaCl_2_, 1 mM MgCl_2_, 2 mM NaHCO_3_, 1.25 mM NaH_2_PO_4_, and 25 mM glucose [19]. The cells were stimulated by applying 100 ng/ml epidermal growth factor (EGF).

Cortical neurons were prepared from newborn Sprague Dawley rats at postnatal day 0 as described previously [20], [21] and cultured in basal medium eagle (BME) supplemented with 10% heat-inactivated bovine calf serum (HyClone, Logan, UT), 35 mM glucose, 1 mM L-glutamine, 100 U/ml penicillin, and 0.1 mg/ml streptomycin. Cytosine arabinoside (2.5 µM) was added to the cultures at days *in vitro* (DIV) 2 to inhibit the proliferation of non-neuronal cells. Cells were transfected at DIV 3 using Lipofectamine 2000 (Invitrogen) as described previously [22]. The cells were imaged at DIV 5–6 in the culture medium, and stimulated with 100 ng/ml brain-derived neurotrophic factor (BDNF).

### 2-Photon Fluorescence Lifetime Imaging Microscopy

A custom-built 2-photon microscope equipped with a Ti:sapphire pulsed laser (MaiTai; Spectra-Physics, Fremont, CA) tuned at 920 nm and a mode-locked Ytterbium-doped laser (1030 nm; Amplitude Systèmes, Bordeaux, France) were used for imaging mEGFP and mRFP distribution, respectively, in 293T cells. The intensity of each laser beam was independently controlled with electro-optical modulators (350-80 LA; Conoptics, Danbury, CT). The two laser beams were combined using a beam-splitting cube and passed through the same set of galvano-scanning mirrors and objective (60×, 0.9 NA; Olympus, Melville, NY). Imaging of dissociated cultures of cortical neurons were performed on a different custom-built two-photon microscope with a Ti:sapphire pulsed laser (MaiTai; Spectra-Physics, Fremont, CA) tuned to 920 nm for imaging of mEGFP- and mRFP-tagged constructs. Fluorescence was divided with a dichroic mirror (565 nm; Chroma) and detected by photomultiplier tubes (PMTs; H7422-40 (Hamamatsu) for green, R3896 (Hamamtsu) for red) after wavelength filters (HQ510/70-2p for green and HQ620/90-2p for red; Chroma Techonology, Brattleboro, VT) [23]. Fluorescence signal was acquired by ScanImage [24] using a data acquisition board (PCI-6110, National Instruments). Fluorescence lifetime images were acquired using a time-correlated single photon counting board (SPC-150; Becker-Hickl) controlled with a custom software integrated into ScanImage [13], [25].

### Fluorescence Lifetime Image Analysis

To generate the fluorescence lifetime image, we calculated the mean photon arrival time, 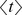, in each pixel as:
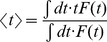
(5)then, the mean photon arrival time is related to the mean fluorescence lifetime, 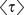, by an offset arrival time, 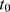, which is obtained by fitting the whole image with Eq. 3:
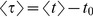
(6)


To quantify Ras activation, the fraction of mEGFP-Ras bound to mRFP-RBD-mRFP was calculated by fitting the fluorescence decay curve summed over the whole image with Eq. 3 [13].

## Results

When 293T cells were transfected with FRas-F (Figure 1C), we observed that mEGFP-HRas was localized at the plasma membrane and internal membranes (Figure 1C), similarly to endogenous HRas [26]. However, we consistently found that the acceptor (mRFP-RBD^R59A^-mRFP) was concentrated in the nucleus, with relatively low expression in the cytosol (Figure 1C). This localization likely limits the sensitivity of the FRas-F sensor by effectively reducing the concentration of RBD in the cytosol. Therefore, we searched for the reason behind the nuclear accumulation of RBD. Upon analysis of the RBD sequence, we detected a nuclear localization sequence (NLS) in RBD (Figure 1B, gray box). To disrupt the NLS, we introduced a K108A mutation in the RBD sequence (Figure 1B, in red). As expected, RBDs with the K108A mutation (RBD^R59A,K108A^) show cytosolic localization (Figure 1C). We named this FRas-F variant FRas2-F. In addition, based on a previous study reporting the affinity of several different RBD mutants [14], we developed another FRas2 variant with an intermediate affinity to HRas (lower than FRas and higher than FRas-F), FRas2-M, by replacing the “F” mutation R59A with K65E (RBD^K65E,K108A^) (Figure 1B).

To further characterize the effect of the K108A mutation on the affinity between Ras and RBD, we measured the affinity between purified sfGFP-HRas without the CAAX membrane targeting sequence and mRFP-RBD or its mutations in the presence of either non-hydrolyzable GTP analog (Gpp(NH)p) or GDP using fluorescence lifetime measurements in a cuvette (Figure 1D; Table 1). We found that the effect of K108A on the affinity of RBD for HRas was relatively minor (RBD^R59A^: 2.0 µM; RBD^R59A,R108A^: 1.7 µM). The affinity of FRas2-M (RBD^K65E,R108A^) was ∼0.5 µM, in between FRas (RBD^WT^; <0.2 µM) and FRas-F, as expected [14].

**Table 1:** Dissociation constants between HRas and RBD or several RBD mutations.

Sensor	Mutations in RBD	HRas-Gpp(NH)p *K*_d_ (µM)	HRas-GDP *K*_d_ (µM)
FRas	None	<0.2	16
FRas-F	R59A	2.0	31
FRas2-F	R59A,K108A	1.7	25
FRas2-M	K65E,K108A	0.5	28

Dissociation constants (*K*_d_) were measured as in Figure 1D (averages of 3–5 independent experiments).

**Figure 2:**
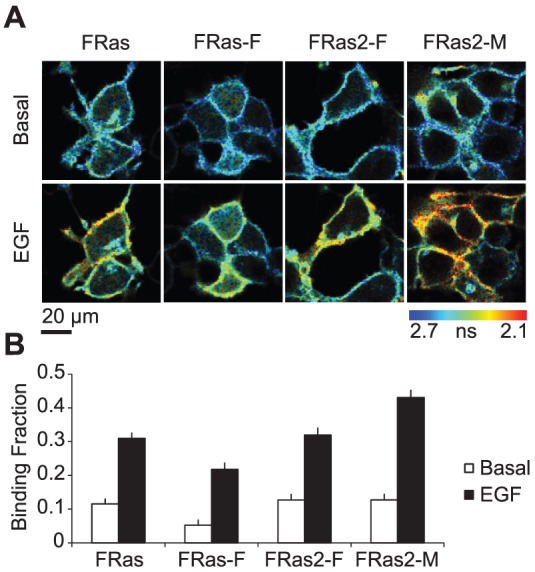
Characterization of FRET sensors for Ras activation in 293T cells. (A) Representative fluorescence lifetime images in 293T cells transfected with Ras sensors, before and after the application of EGF (100 ng/ml). Warmer colors indicate shorter lifetimes and higher levels of Ras activity. (B) Fraction of donor (mEGFP-HRas) bound to acceptor (mRFP-RBD-mRFP) calculated by fitting the fluorescence lifetime curve to a double exponential function, before and after application of EGF. Error bars indicate s.e.m. over 26–34 fields from 3 preparations.

To test the sensitivity of FRas2 variants (FRas2-F and FRas2-M) compared to that of FRas and FRas-F, we transfected these Ras FRET sensors in 293T cells, and imaged them with 2-photon fluorescence lifetime imaging microscopy (2pFLIM) (Figure 2). To activate Ras in 293T cells, we measured Ras activation in response to bath application of EGF [13], [27]. As reported [13], application of EGF (100 ng/ml) increased the binding between mRFP-RBD-mRFP and mEGFP-HRas, indicating that Ras is activated (Figure 2A–B). FRas2-F shows higher binding fraction than FRas-F both before and after EGF stimulation, presumably due to higher acceptor concentration in the cytosol (Figure 2A–B). Further, FRas2-M showed a greater increase in binding fraction after EGF application compared to other sensors (Figure 2A–B). Thus, these results indicate that the new FRas2-M sensor has improved sensitivity.

**Figure 3:**
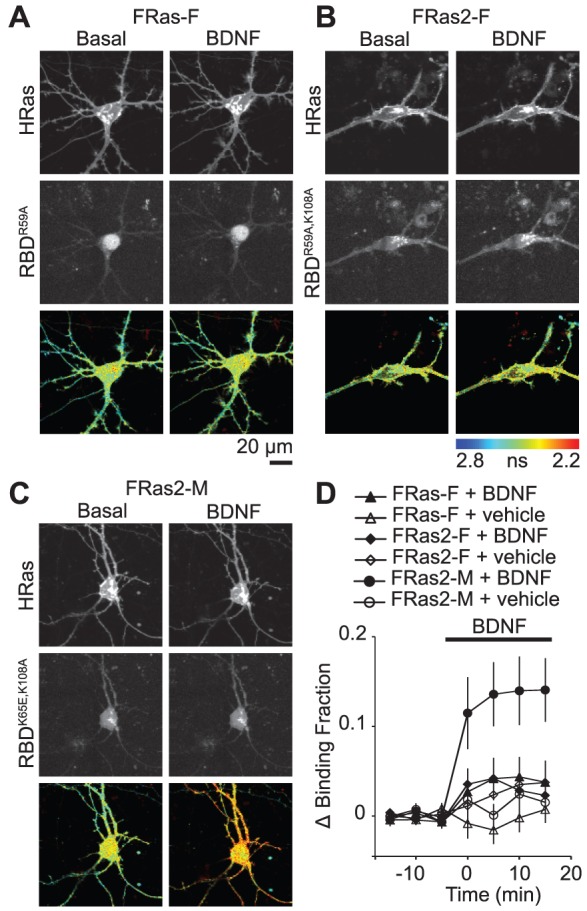
Distribution and characterization of FRET sensors for Ras activation in cortical neurons. (A–C) Representative 2-photon fluorescence images of donor (mEGFP-Ras: top panel) and acceptor (mRFP-RBD-mRFP: middle panel) and fluorescence lifetime images (bottom panel) of FRas-F (A), FRas2-F (B) and FRas2-M (C) in cortical neurons. mRFP images are dim because of the non-optimal excitation wavelength for mRFP (920 nm). (D) Change in the fraction of donor (mEGFP-Ras) bound to acceptor (mRFP-RBD-mRFP) in response to application of BDNF (100 ng/ml). Error bars indicate s.e.m. over 4–11 cells from 3 preparations.

Since Ras signaling is important for many forms of neuronal plasticity and development [1], [6]–[8], we tested the sensitivity of FRas-F, FRas2-F and FRas2-M in neurons. We transfected primary cultures of cortical neurons with these sensors and imaged them with 2pFLIM [13]. The acceptor of FRas-F (mRFP-RBD^R59A^-mRFP) was strongly accumulated in the nucleus (Figure 3A). In contrast, the acceptors of both FRas2-F and FRas2-M were localized to the cytosol and neurites (Figures 3B–C). We observed that Ras activity rapidly peaked after BDNF (100 ng/ml) application, remaining elevated for at least 15 minutes (Figure 3D). Remarkably, FRas2-M showed approximately three fold higher signal compared to the other sensors (Figure 3B–C). These data indicate that FRas2-M has higher sensitivity for reporting Ras activation in neurons compared to FRas2-F and FRas-F.

The sensitivity of the sensor is related to the cytosolic concentration of RBD as well as the binding affinity between Ras and RBD [13]. Thus, we measured the cytosolic concentration of RBD by comparing its fluorescence intensity in the cytosol (mRFP-RBD-mRFP or its mutations) with that of purified mRFP [28]. The concentration was estimated to be 12±3 µM (N = 10) for FRas-F, 30±5 µM (N = 9) for FRas2-F and 24±6 µM (N = 13) for FRas2-M. Since these concentrations are much higher than the dissociation constants of RBDs (Table 1), the simple biochemical scheme does not explain the improvement in sensitivity. It is possible that the effective dissociation constant in cells is much lower than that in solution due to interactions with endogenous proteins. Nonetheless, our results indicate that FRas2-M has much higher sensitivity than other FRas variants.

## Discussion

In this paper, we improved the cytosolic localization of the FRas acceptor by introducing a point mutation to remove the NLS in RBD (Figure 1). Furthermore, we have developed a variant with slightly higher affinity (FRas2-M). The new FRas2-M sensor shows much higher sensitivity in 293T cells and neurons than other FRas variants (Figure 2, 3).

Previously, it has been reported that the inactivation of FRas-F is much faster than FRas due to its lower affinity [13]. Because the affinity of FRas2-M is higher than that of FRas-F, we expect that FRas2-M decays more slowly and thus shows a greater degree of spatial spreading compared to FRas-F (or FRas2-F) [13], [25]. Thus, as with Ca^2+^ indicators, one can use Ras sensors with two different characteristics: a slow sensor with high affinity and high sensitivity (FRas2-M); and fast sensor with low affinity and lower sensitivity (FRas2-F). To quantify the spatiotemporal dynamics of Ras using these sensors, one must measure the dependency of sensor kinetics on RBD concentration and extrapolate to zero expression level [25], [28].

FRas-F has been shown to be useful for imaging Ras activity in single dendritic spines undergoing synaptic plasticity [25]. The improved sensitivity of these FRas2 sensors will provide more detailed information about the spatiotemporal dynamics of Ras in neurons and other cells.
